# Multi-detector computed tomography in the assessment of tetralogy of Fallot patients: is it a must?

**DOI:** 10.1186/s43044-020-00047-3

**Published:** 2020-04-03

**Authors:** Mahmoud Shaaban, Sara Tantawy, Fatma Elkafrawy, Dina Haroun, Soha Romeih, Wesam Elmozy

**Affiliations:** 1 Aswan Heart Centre (Magdi Yacoub Foundation), Aswan, Egypt; 2 Cardiology department, Faculty of medicine, Tanta University, Tanta, Egypt; 3 Radiology department, Faculty of medicine, Ain Shams University, Cairo, Egypt; 4 Radiology department, Faculty of medicine, Alexandria University, Alexandria, Egypt; 5 Radiology department, Faculty of medicine, Cairo University, Giza, Egypt

**Keywords:** Multi-detector computed tomography, Tetralogy of Fallot, Congenital heart disease, MAPCAs, DORV, AVSD, Coronary anomalies, Systemic-to-pulmonary shunts

## Abstract

**Background:**

Tetralogy of Fallot (TOF) accounts for 10% of all CHD. It classically consists of ventricular septal defect (VSD), aortic overriding, right ventricular outflow tract (RVOT) obstruction, and RV hypertrophy. There are many anatomic variants, associated intracardiac and extracardiac anomalies that must be taken into consideration when imaging and planning the surgical procedure needed. Multi-detector computed tomography (MDCT), with its high spatial and temporal resolution, has a pivotal role in the evaluation of complex anatomical findings in both unrepaired and repaired TOF patients.

**Main body:**

Though MDCT has a limited role in the initial diagnosis of TOF, it is particularly important when there is a question about anatomy of pulmonary arteries (PAs) (whether sizable, hypoplastic, or atretic), presence of major aorto-pulmonary collaterals (MAPCAs) and presence of additional VSDs. Additionally, MDCT is crucial in the diagnosis of different anatomical variants of TOF. TOF patients with absent pulmonary valve classically have hugely dilated PAs which raise an important question about the degree and severity of airways compression. This question can be accurately answered by MDCT. TOF with double-outlet RV (DORV) has variable degrees of aortic override which can be assessed by MDCT. An atrio-ventricular septal defect (AVSD) is seen in about 13% of TOF cases and typically occurs in patients with Down syndrome. MDCT can assess the size and extent of inlet VSD and size of both ventricles (balanced or unbalanced AVSD). Coronary artery anomalies are common and important association. MDCT can identify the presence of a major coronary artery crossing the RVOT, a left anterior descending (LAD) from RCA, or a dual LAD. The clinical importance of these anomalies is its susceptibility to injury during ventriculotomy incision required for TOF repair necessitating changing the usual approach of surgery.

Patients with reduced pulmonary blood flow undergo a systemic to pulmonary shunt. MDCT can assess the patency of the shunt, stenotic, or occluded segments. In surgically repaired TOF patients, MDCT can identify the sequalae and long-term complications including residual RVOT obstruction, conduit stenosis, RVOT patch aneurysm, RVH, and aortic root dilatation.

**Conclusion:**

MDCT is a safe and reliable imaging modality that provides accurate assessment of anatomical variants and associated anomalies of TOF.

## Background

Tetralogy of Fallot (TOF) is the most common cyanotic congenital heart disease (CHD). In neglected untreated cases, the mortality rate is about 33% in the first year of life and about 50% in the first 3 years of life [1]. However, advances in diagnosis, surgical techniques, and postoperative treatment have led to increasing number of patients reaching adulthood with a dramatic increase in survival rate to almost 90% at 30 years. TOF was originally named after the French scientist “Arthur Louis Etienne Fallot” who published a paper in 1988 entitled “Contribution a ‘l’anatomie pathologique de la maladie bleue” [2]. He described four anatomical features that were almost always present in all post-mortem specimens of the blue patients “la maladie bleue” in his own words. These anatomical features consist of a tetrad of (1) ventricular septal defect (VSD), (2) aortic overriding, (3) right ventricular outflow tract (RVOT) obstruction, and (4) right ventricular (RV) hypertrophy [3].

### Pathophysiology

The main pathophysiological features of TOF are the antero-cephalic deviation of the cono-ventricular septum and hypertrophy of the septoparietal trabeculations of the RVOT [3]. The antero-cephalic deviation of the cono-ventricular septum results in a mal-aligned VSD in continuity of the aortic valve [4]. Thus, the aortic valve has a biventricular connection with variable degrees of overriding the RV. In addition, the mal-alignment between the cono-ventricular septum and the septoparietal trabeculations of the RVOT results in hypertrophy of the RVOT and subsequent pulmonary valvular or infundibular stenosis [5–8]. The hemodynamic effect of RVOT obstruction is right ventricular hypertrophy which is the fourth pathological feature of TOF [3].

### Clinical presentation and diagnosis

Although TOF is the most common cyanotic congenital heart disease, only a small percentage of cases are deeply cyanotic since birth after closure of the ductus arteriosus. This group of cyanotic patients is referred to as “blue TETs” while the acyanotic group is referred to as “pink TETs”. Blue TETs typically have severe pulmonary stenosis or atresia. Sometimes, these patients may remain undiagnosed in the first days or weeks of life due to the presence of moderate to large patent ductus arteriosus (PDA) which maintains adequate blood flow to lungs and present with profound cyanosis after duct closure necessitating emergency surgery to maintain adequate pulmonary blood flow.

Pink TETs may have mild desaturation with arterial oxygen saturation in the range of 80–90% or even normal oxygen saturation depending on the degree of pulmonary stenosis and the presence of PDA or major aorto-pulmonary collateral arteries (MAPCAs). Usually, the baseline oxygen saturation gradually decreases as the right ventricular hypertrophy (RVH) progress and subsequently the degree of RVOTO. These patients should be closely followed up to determine the appropriate time of surgical intervention before cyanosis worsens. Pink TETs can develop episodic profound hyper-cyanosis “cyanotic spells” with arterial oxygen saturation drop to below 50% and manifest by sudden marked increase in cyanosis, shortness of breath, agitation, and loss of consciousness. These cyanotic spells result from dynamic changes in the pulmonary and systemic resistance which is thought to be due to infundibular muscle spasms with large dynamic increase in RVOT obstruction resulting in severe right to left shunting of blood through the VSD [9].

Clinical examination of TOF patients can reveal variable degrees of cyanosis, ranging from mild to severe generalized cyanosis. Clubbing may be seen in neglected cases. On auscultation, an ejection systolic murmur can be heard due to RVOT obstruction which usually soften or disappear during cyanotic spells as a result of marked decrease of blood flow across RVOT. The VSD typically has no appreciable murmur because of its large size and systemic level pressure in the RV. Usually, there is no splitting of second heart sound because of small pulmonary valve and low post-stenotic pulmonary artery pressure [9]. Electrocardiogram shows right ventricular hypertrophy and right axis deviation.

Classical finding in chest radiography is the “boot-shaped” heart resulting from RVH and small pulmonary knob and the lung is usually oligemic due to decreased pulmonary blood flow.

Echocardiography is the diagnostic modality of choice. It provides complete evaluation of the anatomy, estimation of pressure gradient across the RVOT, identification of the level of RVOT obstruction, and delineation of coronary artery anatomy [10]. Prenatal echocardiography is highly accurate in the in utero diagnosis of TOF, although the degree of pulmonary stenosis is usually difficult to be determined. More data are needed to identify the effect of prenatal diagnosis on long-term survival [11]. Three-dimensional echocardiography is not yet superior to two-dimensional echocardiography in diagnosis and preoperative assessment of TOF, though it may be of great value in long-term follow-up of functional and morphological changes to the RV in repaired TOF patients [12].

Before the advanced techniques in echocardiography, cardiac catheterization was used to be the gold standard for the diagnosis of TOF. However, in the current days, the use of cardiac catheterization is limited and preserved only for cases with unclear anatomy of branch pulmonary arteries and to confirm the presence of anomalous coronary artery. As a good alternative to invasive cardiac catheterization, three-dimensional computed tomography and magnetic resonance imaging play an important role in the evaluation of TOF anatomy. These modalities can accurately reveal branch pulmonary arteries, aortic arch anatomy, and also delineate coronary anomalies.

Cardiac magnetic resonance (CMR) is currently considered as one of the most used modalities for long-term evaluation of repaired TOF patient. CMR measurements of RV volumes and function and accurate quantification of pulmonary valve regurgitation volume and fraction, in addition to monitoring the progress of pulmonary regurgitation and RV dilatation, are superior to those obtained by two-dimensional and three-dimensional echocardiography [13, 14]. These CMR measurements are becoming very dependable indications for timing of re-interventions and most importantly the timing of pulmonary valve replacement [15].

In addition to standard CMR protocols, time-resolved 3D phase contrast MRI (also called 4D flow MRI) improves the visualization and quantification of blood flow in the cardiovascular system that helps in obtaining more detailed information about vascular hemodynamics. Thus, this technique may be a promising method to accurately evaluate altered blood flow in patients with CHD with complex anatomy and hemodynamics.

### Medical management

There is no definitive medical management of TOF patients, and the only definitive management is surgical repair. However, there are few significant medical adjuncts, the most important of which is the Prostaglandin E1 (PGE1) which is used in profoundly cyanosed neonates to maintain the patency of ductus arteriosus [16]. PGE1 is given as a continuous intravenous infusion at a dose of 0.01–0.1 μg/kg/min, to maintain ductal patency, thus providing maintained pulmonary blood flow until definitive surgical correction or palliative surgery by establishment of systemic to pulmonary shunt. Administration of PGE1 is mainly limited to the first week of life, after which PGE1 rarely results in duct opening. The most important and significant side effect of PGE1 is apnea which may require endotracheal intubation and mechanical ventilation.

Other medical adjuncts are mainly aiming to prevention or treatment of cyanotic spells. Beta-blockers may be used in prevention of these spells by reducing the muscular spasm in the RVOT infundibulum [17, 18]. However, it has been shown that TOF patients receiving beta-blockers are at increased risk of inotropes dependence and temporary pacing following surgical repair [19]. Thus, earlier surgical management is recommended in patients with paroxysmal spells requiring beta-blockers.

Acute management of cyanotic spells includes placing the patient in knee head position, proper oxygenation, sedation, volume resuscitation, and alpha-receptor antagonists like phenylephrine which cause systemic vasoconstriction, increase systemic vascular resistance, decrease right to left shunting of the blood across the VSD, and thus increase the pulmonary blood flow [20]. However, any TOF patient requiring such therapy should be referred for urgent surgical management.

### Surgical options

Before the introduction of surgical interventions for TOF, the mortality was about 35% of patients at the first year of life, 50% at the age of 3 years and the survival after the age of 30 years was rare [21]. Nowadays, almost all patients that undergo surgical correction are expected to reach adult life.

The best timing for elective surgical repair is now considered to be within the first year of life [22, 23]. Early surgical repair before the age of 3 months has been reported to be associated with extended intensive care stay and hospitalization [22], So such an early repair should be reserved only for patients with severe cyanosis or cyanotic spells. Late surgical correction after the age of 1 year may be associated with complications of long-lasting right ventricle pressure overload and cardiomyopathy due to long-term hypoxemia, which has been related to ventricular dysfunction and arrhythmias [22].

### Complete surgical repair

The aim of the corrective surgery is to completely close the VSD and relieve the RVOT obstruction, with preservation of the right ventricular and pulmonary valve functions. Surgical approach during the early years of corrective surgery was through right ventriculotomy. However, from the mid-sixties, transatrial-transpulmonary approaches with or without patch repair of the outflow tract improved early to middle-term outcome [24]. Currently, surgical correction of TOF may consist of complete relief of RVOT obstruction with extensive resection of infundibular muscles, but often at the expense of pulmonary valve regurgitation, or sometimes accepting residual obstruction in order to preserve pulmonary valve function, aiming to minimizing late surgical sequalae [23].

### Palliative procedures

Palliative procedures are usually performed in patients who are unfit or have contraindications to corrective surgery including aberrant coronary arteries, hypoplastic or small caliber pulmonary arteries, and associated cardiac malformations. The aim of palliative surgery is to increase the pulmonary blood flow so as to promote the growth of pulmonary arteries in preparation for corrective surgery at second stage. Various types of palliative procedures have been developed over time. The current palliative surgery procedure of choice is the modified Blalock-Taussig (MBT) shunt, where a Gore-Tex graft is placed between one of the arch vessels and the pulmonary artery. Typically, the shunt is placed on the opposite side of the aortic arch; thus, a patient with a left sided aortic arch receives a right sided MBT shunt. This shunt increases pulmonary blood flow and helps the pulmonary arteries to grow [25].

## Multi-detector computed tomography in tetralogy of Fallot

Non-invasive cardiac imaging plays a critical role in the initial diagnosis and follow-up of TOF patients. Echocardiography is the initial modality of choice for making the diagnosis and follow-up. However, multi-detector computed tomography (MDCT), with its high spatial and temporal resolution, provides detailed depiction of cardiac anatomy and morphology and thus has a pivotal role in the evaluation of complex anatomical findings in both unrepaired and repaired TOF patients. In addition, performing and interpreting cardiac CT examination in unrepaired TOF is essential to guide the surgical intervention needed for repair.

### The anatomy of pulmonary arteries

**Fig. 1:**
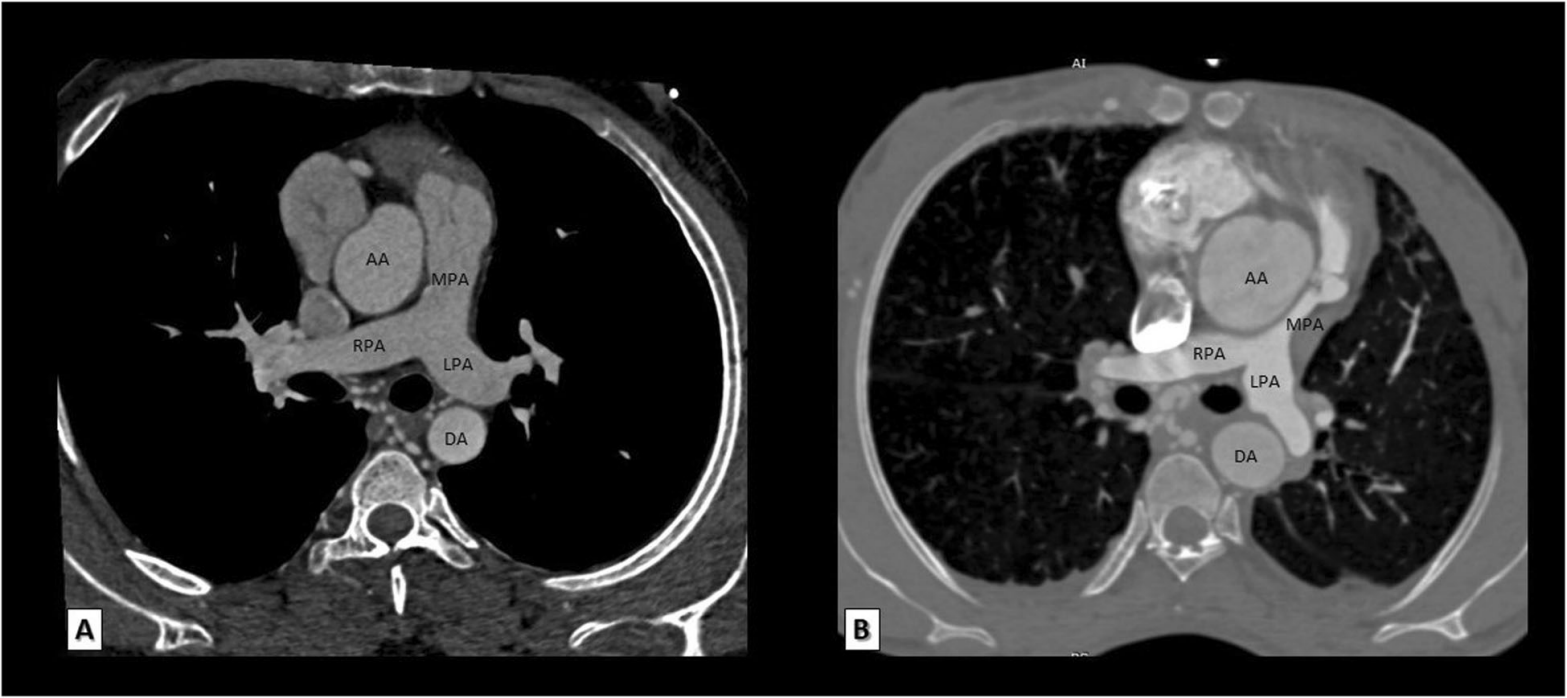
Reformatted MDCT axial images of two different patients. The first patient with sizable main pulmonary artery as well as its right and left pulmonary branches (**a**) and the second patient with sizable right and left pulmonary arteries but with hypoplastic main pulmonary artery (**b**). AA, ascending aorta; MPA, main pulmonary artery; RPA, right pulmonary artery; LPA, left pulmonary artery; DA, descending aorta

MDCT is particularly important when there is a question about anatomy of pulmonary arteries. The main pulmonary artery and/or one of its right and left branches may be sizable, hypoplastic, or even atretic (Fig. 1). This is critical to identify because patients with hypoplastic or atretic pulmonary arteries will undergo a palliative procedure while patients with sizable pulmonary arteries may be good candidates for corrective surgery.

### Major aorto-pulmonary collateral arteries (MAPCAs)

**Fig. 2:**
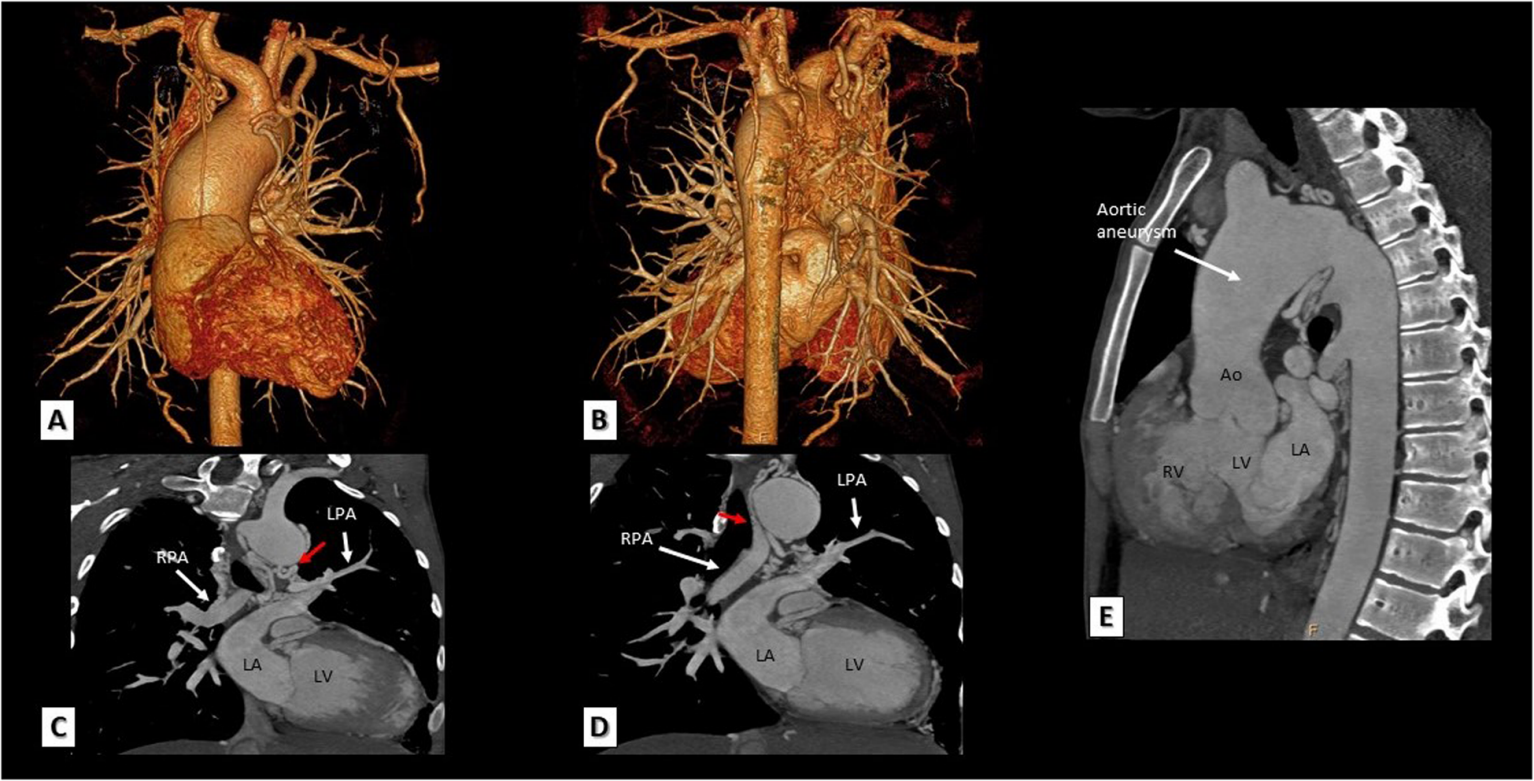
A neglected adult case with TOF and pulmonary atresia. **a**, **b** 3D VRT MDCT images showing innumerous MAPCAs filling the mediastinum arising from descending thoracic aorta as well as right and left subclavian and internal mammary arteries. **c**, **d** Reconstructed MDCT coronal images showing pulmonary atresia and non-confluent right and left pulmonary arteries arising from MAPCAs rather than main pulmonary artery (red arrows). **e** A reconstructed MDCT sagittal image showing ascending aortic aneurysm as a result of long-standing volume loading in this patient. Ao, aorta; RPA, right pulmonary artery; LPA, left pulmonary artery; LV, left ventricle; RV, right ventricle; LA, left atrium.

The majority of these systemic to pulmonary collaterals originate from the descending thoracic aorta; however, they may also originate from the aortic arch, distal thoracic aorta, subclavian, and internal mammary or left coronary arteries (Fig. 2). The modality of choice to identify these MAPCAs is the MDCT and it is critical to identify because these MAPCAs must be closed either percutaneously or surgically during complete corrective surgery in order to avoid pulmonary over flow after surgical correction.

### Additional ventricular septal defects (VSD)

The VSD is a sequel of antero-cephalic deviation of cono-ventricular septum. The most common type is the peri-membranous type which occurs in about 80% of cases. The VSD is typically large and can be identified easily by echocardiography. However, this cono-ventricular VSD may be accompanied by additional VSDs with an incidence of 5% [26]. These VSDs are usually small muscular, requiring special attention as they may be sometimes difficult to identify by echocardiography because the equalized LV and RV pressures—as a result of the large cono-ventricular VSD—make no significant pressure gradient across these additional VSDs. Here comes the crucial role of MDCT in identifying these additional VSDs which—if present—must be closed during surgical repair to avoid pulmonary over flow after surgical correction.

### MDCT in identification of different anatomic variants of TOF

#### TOF with atrio-ventricular septal defect (AVSD)

**Fig. 3:**
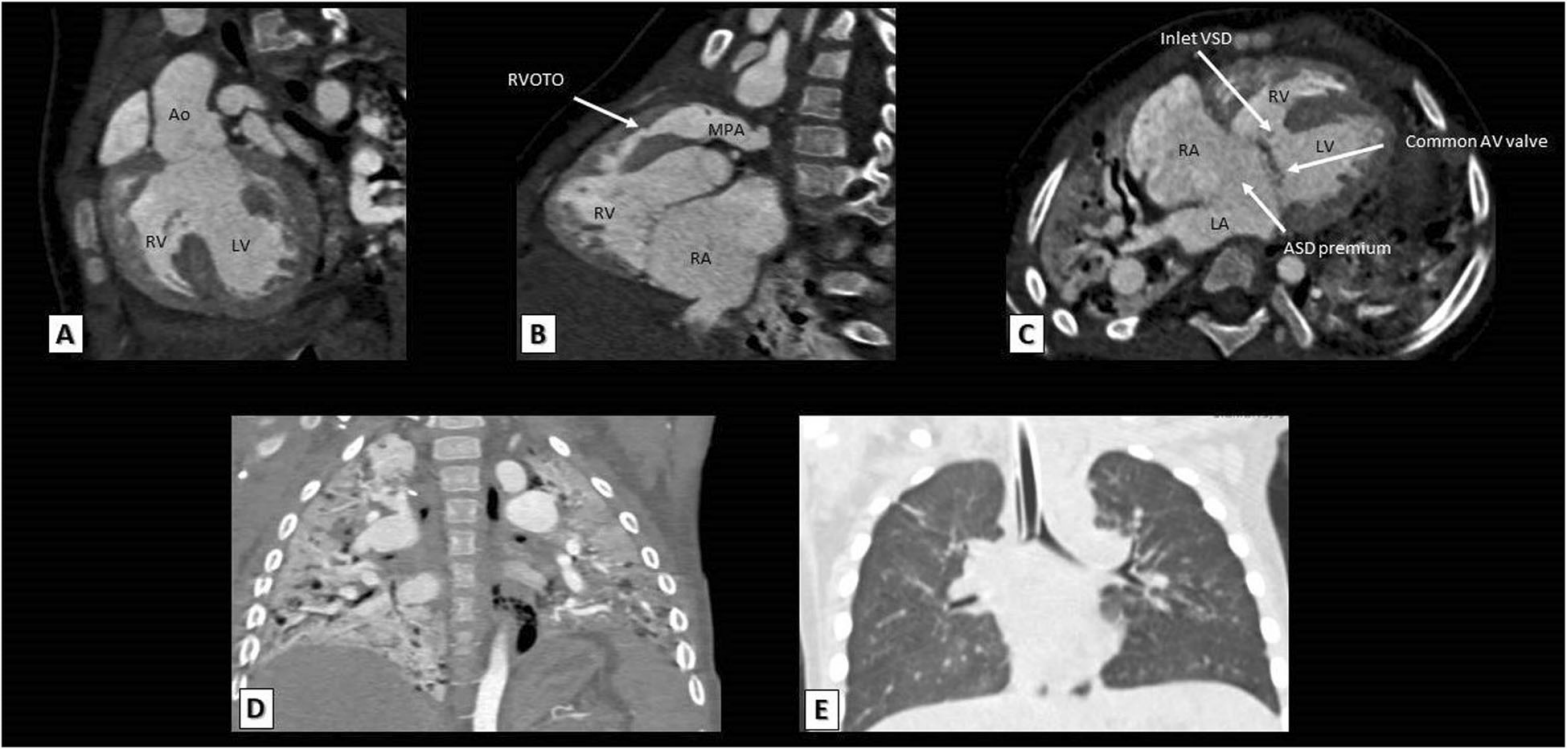
**a**, **b**: Reconstructed MDCT coronal and sagittal images showing overriding of aorta over the VSD (**a**), RVOT obstruction and RV hypertrophy (**b**). **c** A reformatted MDCT axial image showing a complete AVSD with ostium premium ASD, a common AV valve, and an outlet VSD. **d** Bilateral transient total lung collapse as a result of airway obstruction during anesthesia, which completely resolved minutes later after airway tube insertion (**e**). Ao, aorta; LV, left ventricle; RV, right ventricle; LA, left atrium; RA, right atrium; MPA, main pulmonary artery; RVOTO, right ventricular outflow tract obstruction

An AVSD is seen in about 13% of TOF cases [27] and TOF with associated AVSD occurs typically, but not exclusively, in patients with trisomy 21 “Down syndrome” [28]. The rule in such cases is a common AV valve “complete type”, but rarely, it may associated with two separate AV valves “Intermediate type” [29]. MDCT can assess the size and extent of inlet VSD and also the size of both ventricles to determine whether this is a balanced or unbalanced AVSD (Fig. 3). This is pivotal in the decision of surgery because TOF patients with balanced AVSD may undergo complete surgical repair while patients with unbalanced AVSD may undergo single ventricle palliation.

#### TOF with associated coronary anomalies

**Fig. 4:**
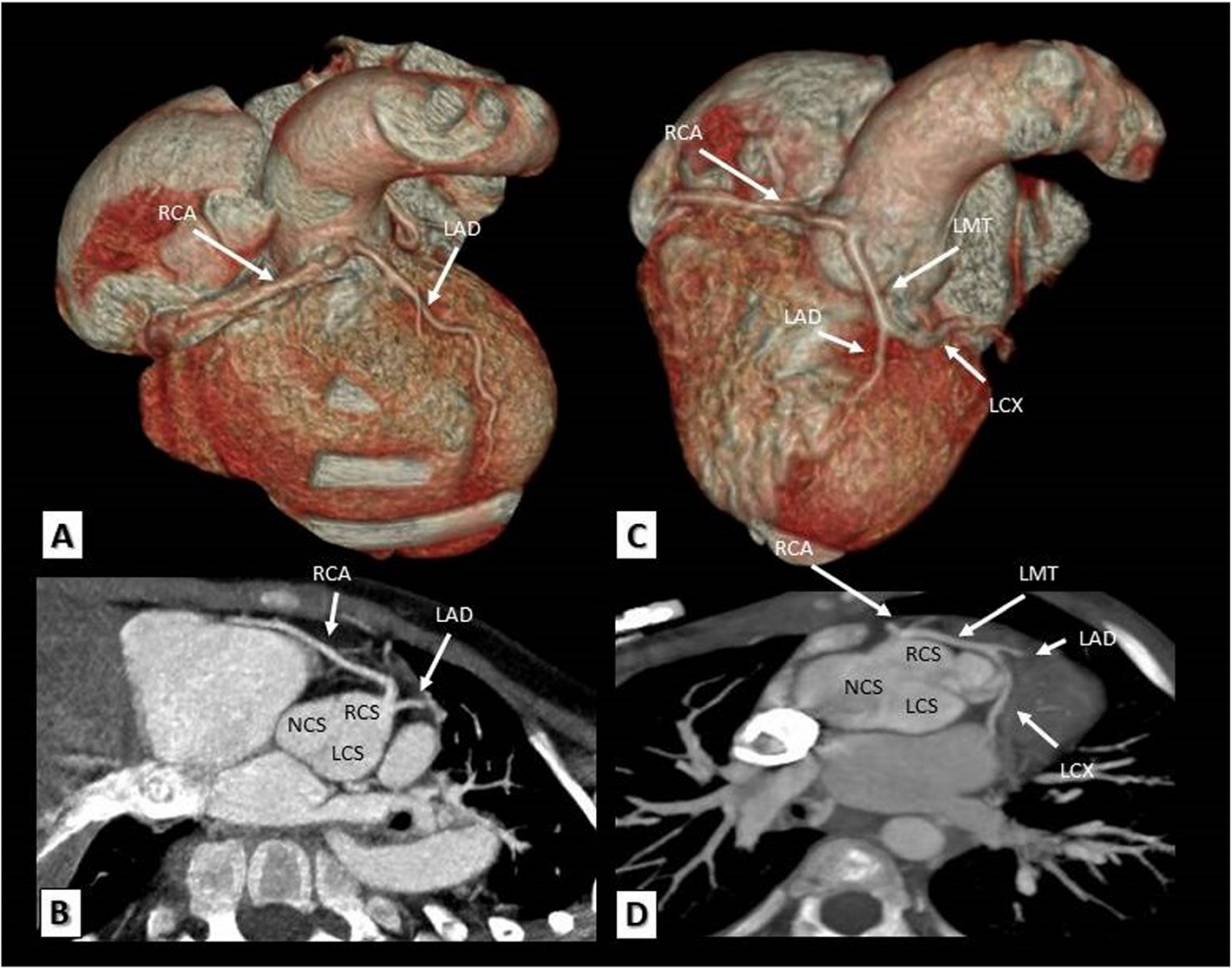
**a**, **b** A TOF patient. **a** 3D VRT and **b** reformatted MDCT axial images showing the left anterior descending artery arising from the right coronary sinus and crossing the RVOT anteriorly to reach the anterior inter-ventricular groove. **c**, **d** Another TOF patient. **c** A VR and **d** a reformatted MDCT axial images showing a long left main trunk originating from the right coronary artery and crossing the RVOT anteriorly before it bifurcates to left anterior descending and left circumflex coronary arteries. LAD, left anterior descending; RCA, right coronary artery; LMT, left main trunk; LCX, left circumflex coronary artery; RCS, right coronary sinus; LCS, left coronary sinus; NCS, non-coronary sinus

**Fig. 5:**
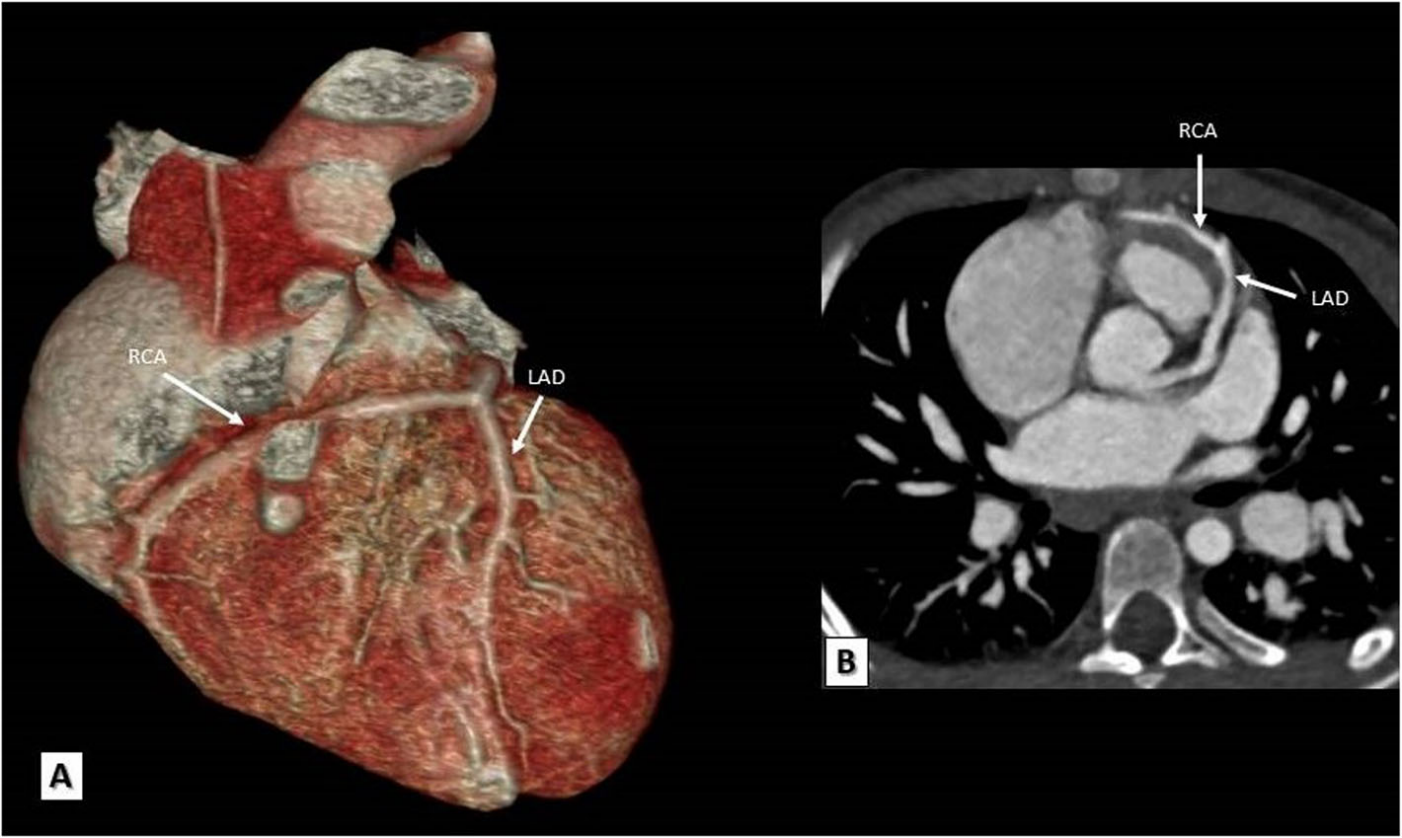
**a**, **b** A TOF patient. **a** 3D VRT and **b** reformatted MDCT axial images showing the right coronary artery arising from the left anterior descending artery and crossing the RVOT anteriorly to reach the right atrio-ventricular groove. LAD, left anterior descending; RCA, right coronary artery

An important association with TOF is coronary artery anomalies, the most frequent of which is a major coronary artery passing anteriorly and crossing the RVOT which accounts for approximately 5–12% of TOF cases. The most frequently and significantly involved major coronary artery is the left anterior descending (LAD) coronary artery originating from the right coronary artery (RCA) and crossing anteriorly the subepicardial surface of the RVOT shortly below the pulmonary annulus to reach the anterior inter-ventricular groove. The clinical importance of this anomalous LAD artery is its susceptibility to injury during ventriculotomy incision required for TOF repair necessitating changing the usual approach of surgery [30]. Less commonly, a dual LAD coronary artery originating from both the RCA and the left main (LM) coronary artery may occur. Rarely, all coronary arteries may originate from a single ostium within the right sinus of Valsalva. Sometimes, one or two accessory coronary ostia can be detected within the right sinus of Valsalva giving origin to one or more conal branching also crossing the RVOT anteriorly [30]. These branches are only considered significant and represent a contraindication to ventriculotomy if their calibers are equal to or larger than the caliber of RCA. MDCT can reliably identify all forms of coronary anomalies and guide the surgical approach for TOF patients (Figs. 4 and 5).

#### TOF with atrial septal defect (ASD)

**Fig. 6:**
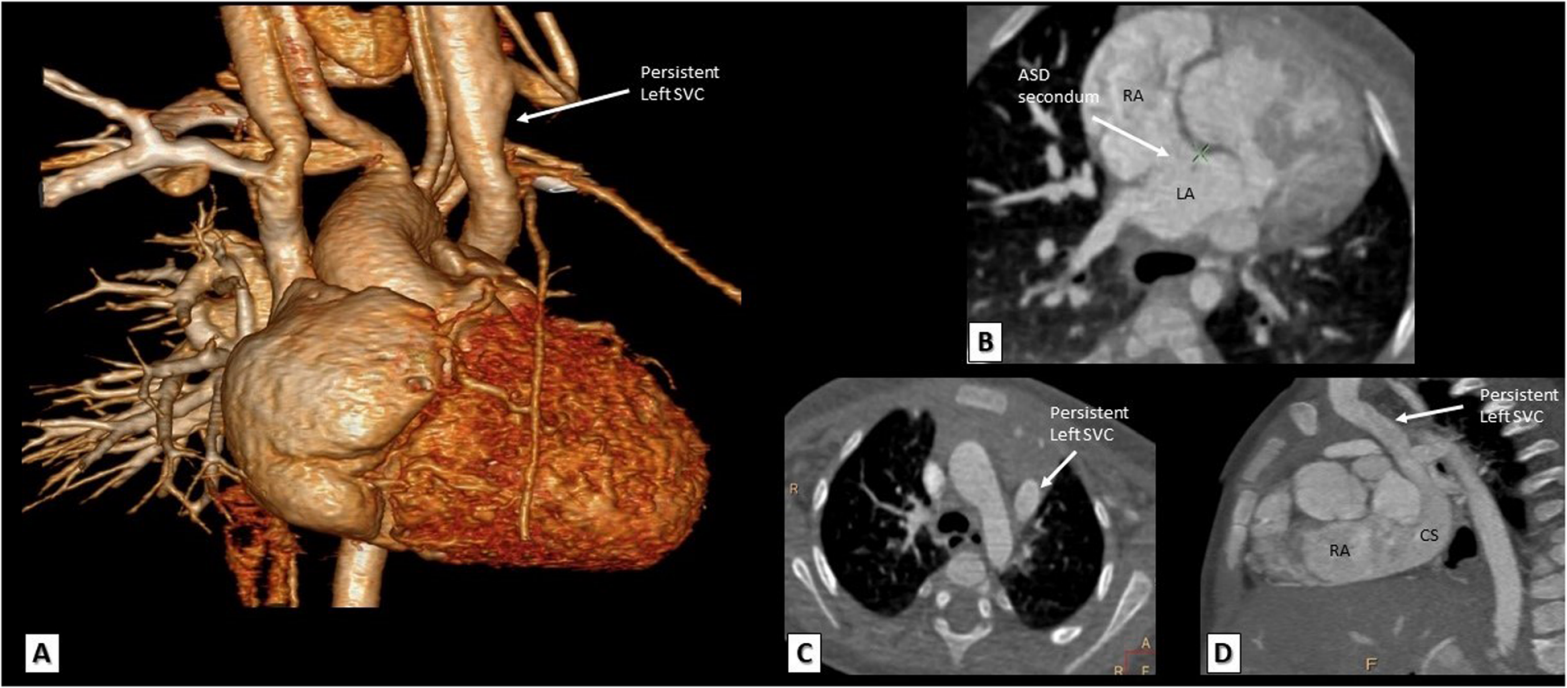
**a** A 3D VRT. **b**, **c** Reformatted axial and **d** reconstructed sagittal MDCT images. This TOF patient has an associated ostium secondum ASD (**b**) in addition to persistent left superior vena cava draining into the coronary sinus (**a**, **c**, **d**). ASD, atrial septal defect; SVC, superior vena cava; RA, right atrium; CS, coronary sinus; LA, left atrium

A patent foramen ovale “PFO” or an ostium secondum ASD is a common association with TOF and is known as pentalogy of Fallot. The size and extent of this defect can be easily and surely identified by MDCT (Fig. 6).

#### TOF with absent pulmonary valve

**Fig. 7:**
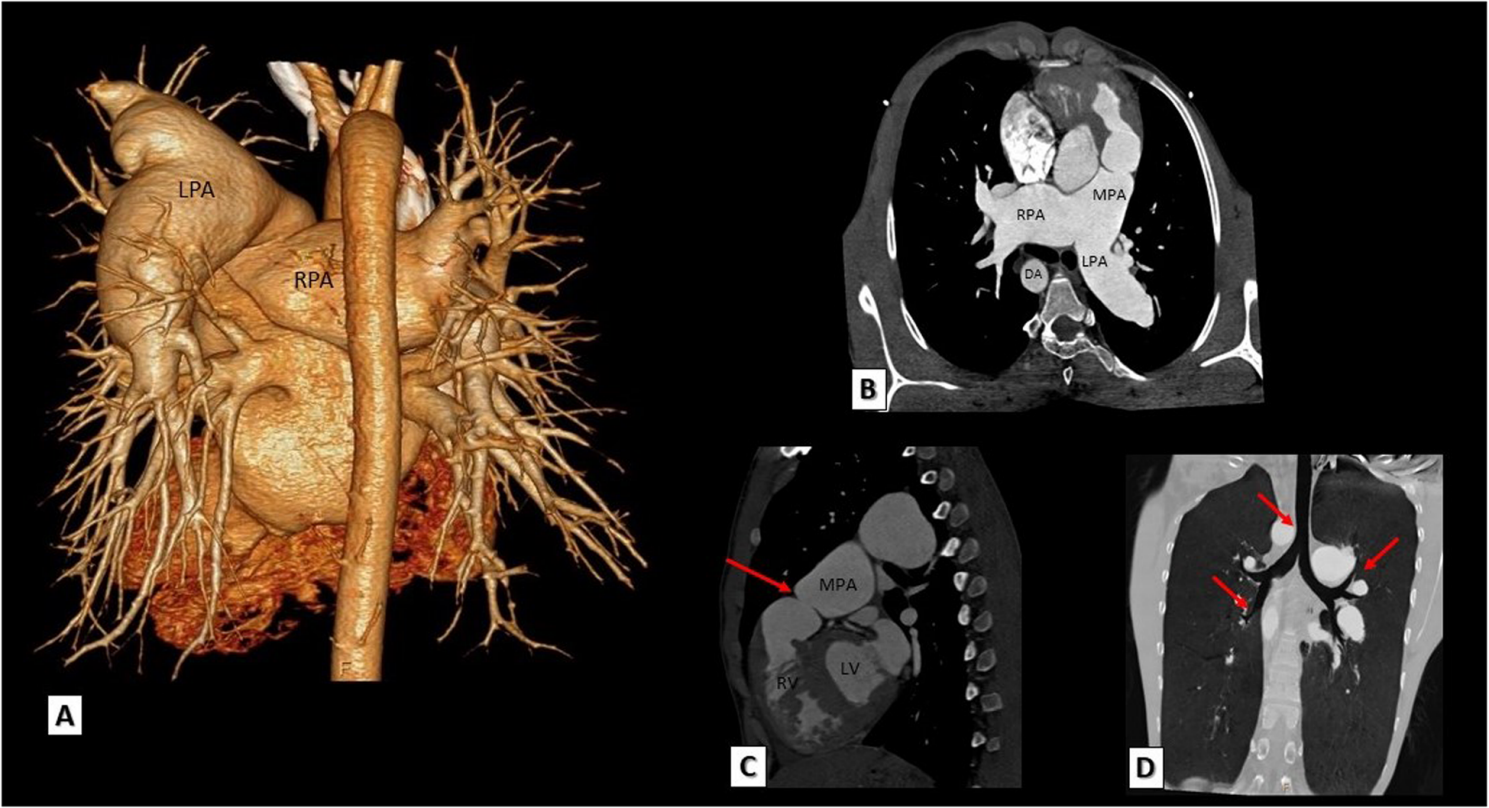
A case of TOF with absent pulmonary valve. **a** 3D VRT and **b** reformatted axial MDCT images showing hugely dilated pulmonary arteries. Note the associated right sided aortic arch. **c** A reconstructed sagittal MDCT image showing absent pulmonary valve (red arrow) and RVOT obstruction. **d** A reconstructed coronal MDCT MIP image showing mild compression on trachea and airway (red arrows). RPA, right pulmonary artery; LPA, left pulmonary artery; MPA, main pulmonary artery; DA, descending aorta; LV, left ventricle; RV, right ventricle

TOF patients with absent pulmonary valve classically have hugely dilated pulmonary arteries which raise an important question about the degree and severity of airways compression. The lower trachea and right and/or left main bronchus can be affected. Prolonged external compression may lead to tracheomalacia or bronchomalacia. The degree of airway compression can be accurately assessed by MDCT (Fig. 7).

#### TOF with double-outlet right ventricle (DORV)

**Fig. 8:**
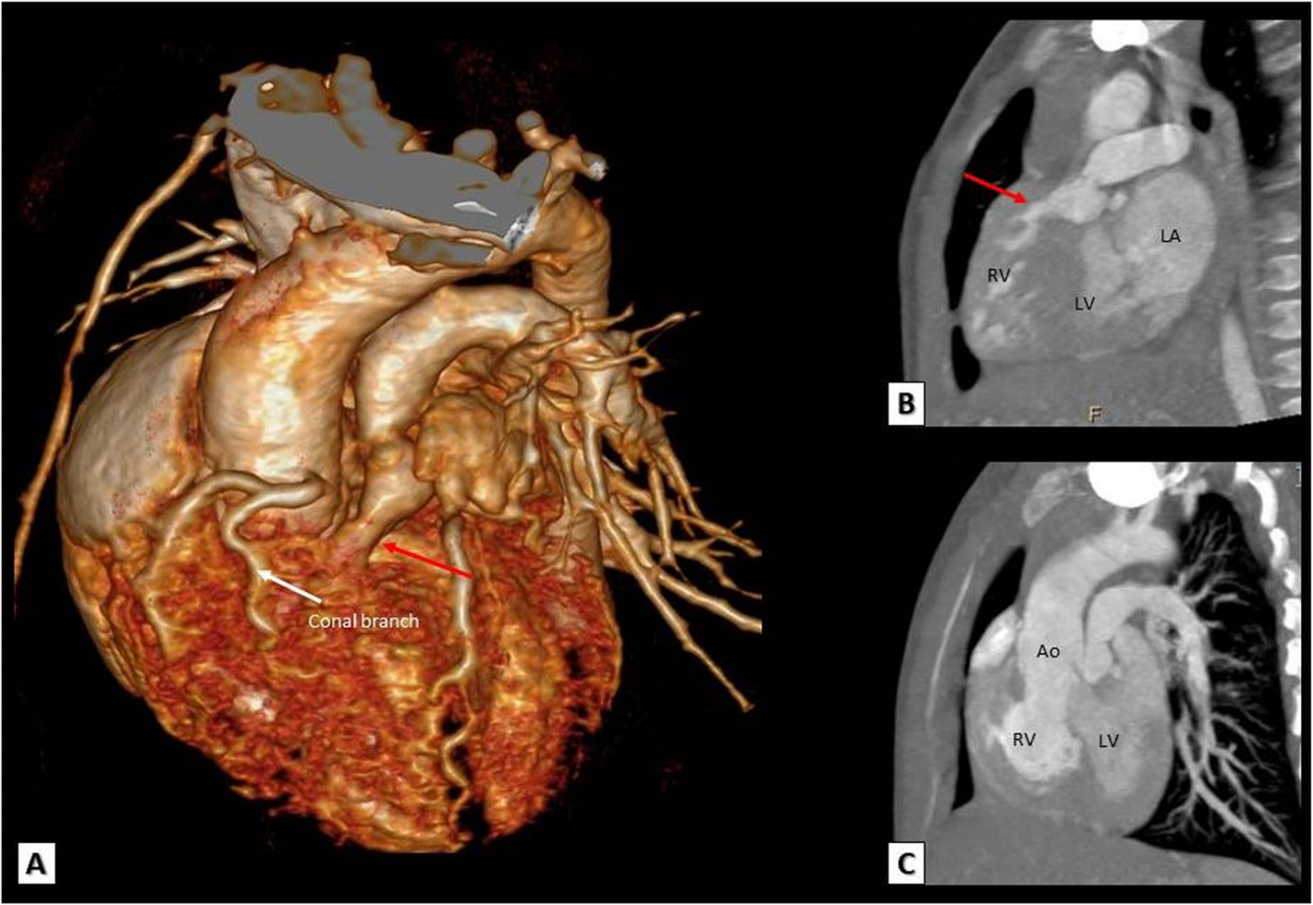
**a** A 3D VRT. **b** Reconstructed coronal and **c** reconstructed sagittal MDCT images showing a double-outlet right ventricle where the pulmonary artery and more than 50% of the aorta originate from the right ventricle. Note the RVOT obstruction (red arrows) and a significant conal branch crossing the RVOT that should be avoided during surgical incision. Ao, Aorta; LV, left ventricle; RV, right ventricle; LA, left atrium

TOF is associated with variable degrees of aortic override. The mode of ventriculo-arterial connection may be concordant when more than 50% of the aorta is supported by the LV or double-outlet ventricular connection may occur when more than 50% of the aorta is supported by the RV. This is the DORV variant of TOF. The degree of aortic override can be easily detected by MDCT (Fig. 8).

#### MDCT in TOF patients after palliative procedures

**Fig. 9:**
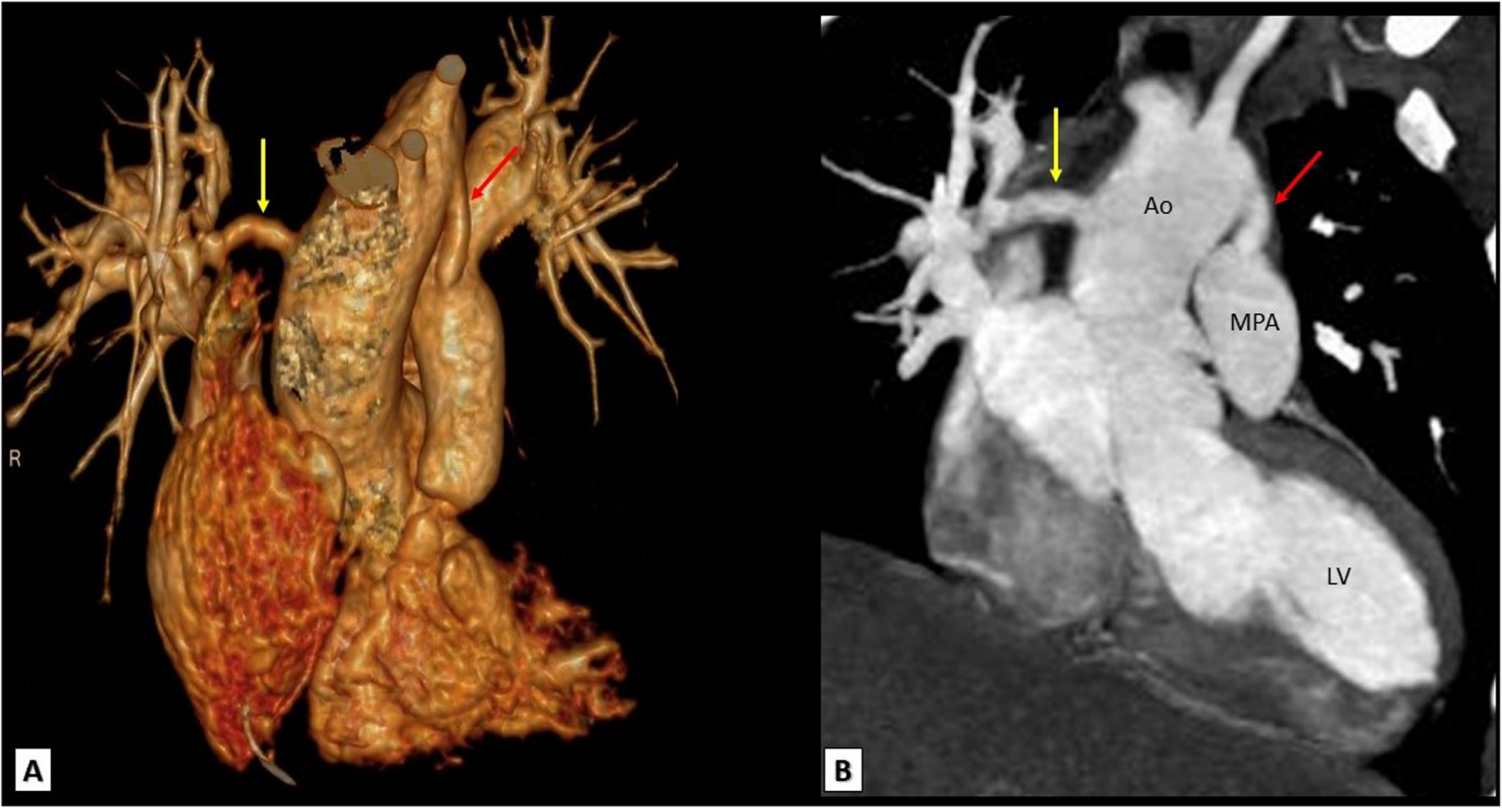
A TOF patient with bilateral central shunts. **a** 3D VRT and **b** reconstructed coronal MDCT images showing a patent central shunt between the aortic arch and main pulmonary artery (red arrows) and another patent central shunt between the ascending aorta and distal right pulmonary artery (yellow arrows). Ao, Aorta; MPA, main pulmonary artery; LV, left ventricle

**Fig. 10:**
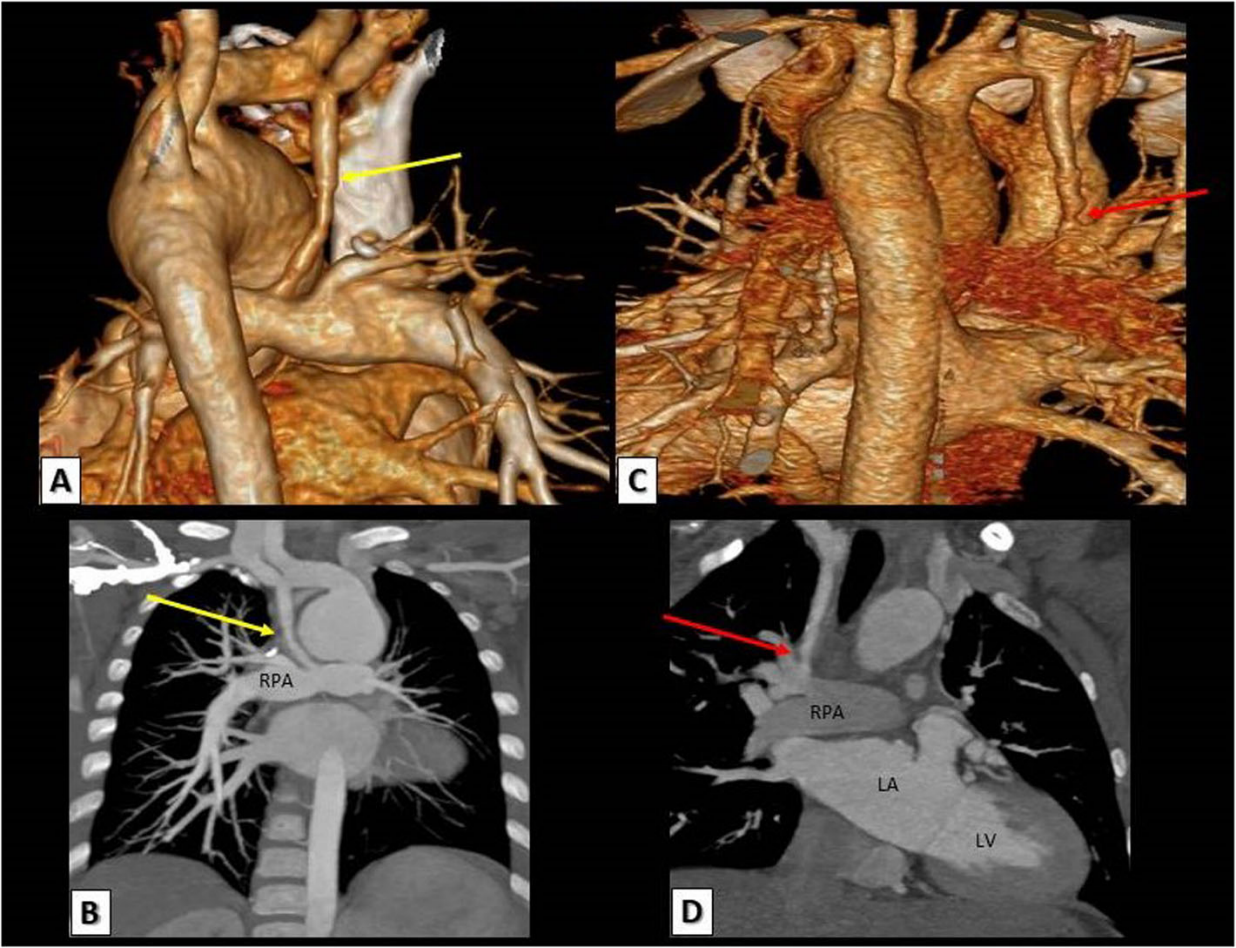
a, b A TOF patient with right MBT shunt. **a** 3D VRT and **b** reconstructed coronal MDCT images showing a patent MBT shunt between the right subclavian artery and right pulmonary artery. **c**, **d** Another TOF patient with also right MBT shunt. **c** 3D VRT and **d** reconstructed coronal MDCT images showing a significant stenosis at the pulmonary end of the MBT shunt. RPA, right pulmonary artery; LA, left atrium; LV, left ventricle

**Fig. 11:**
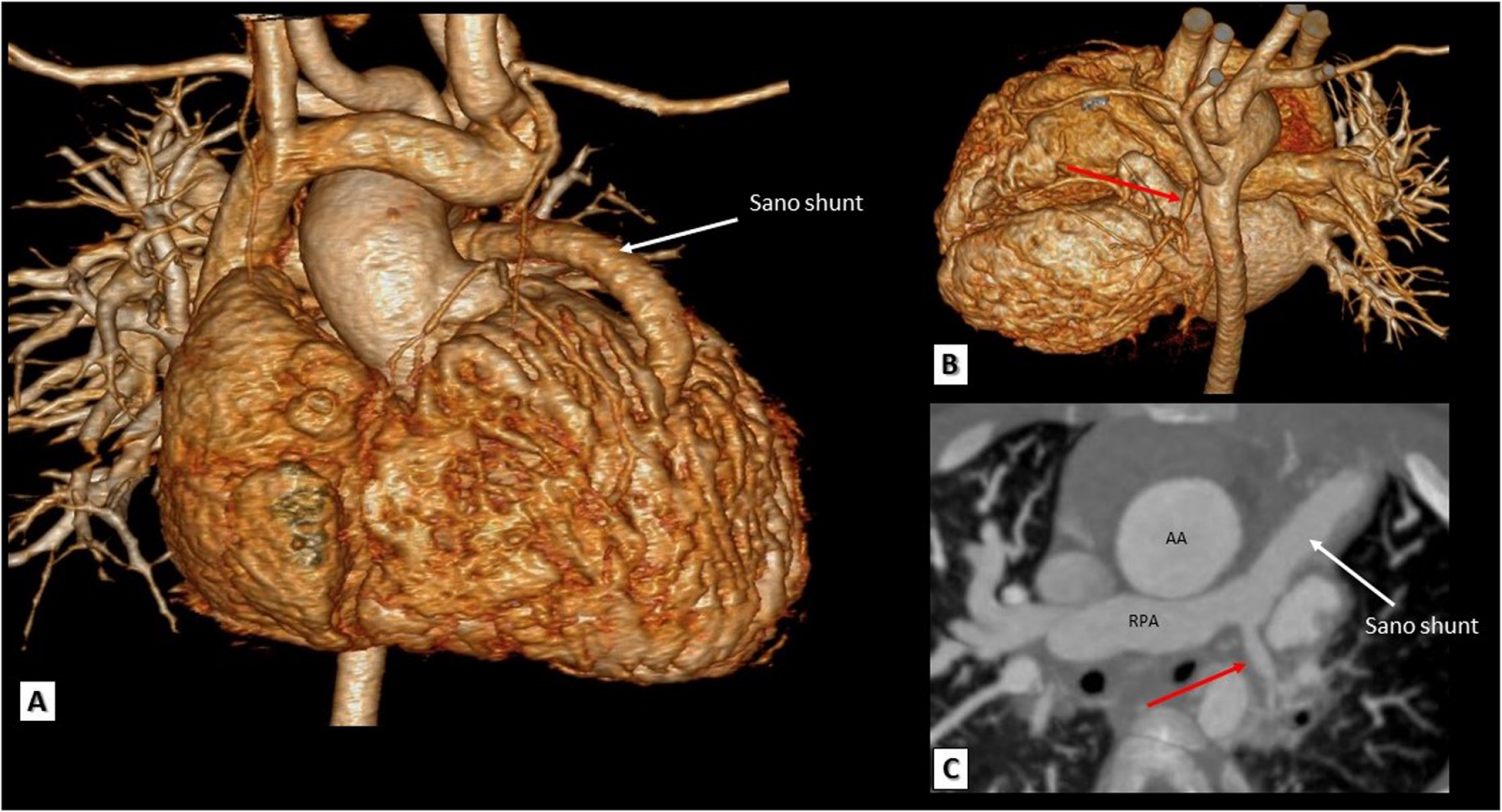
A TOF patient with Sano shunt. **a**, **b** 3D VRT and **c** a reformatted axial MDCT image showing a patent Sano shunt between the RV and the distal main pulmonary artery. Note the hypoplastic left pulmonary artery (red arrows). AA, ascending aorta; RPA, right pulmonary artery

**Fig. 12:**
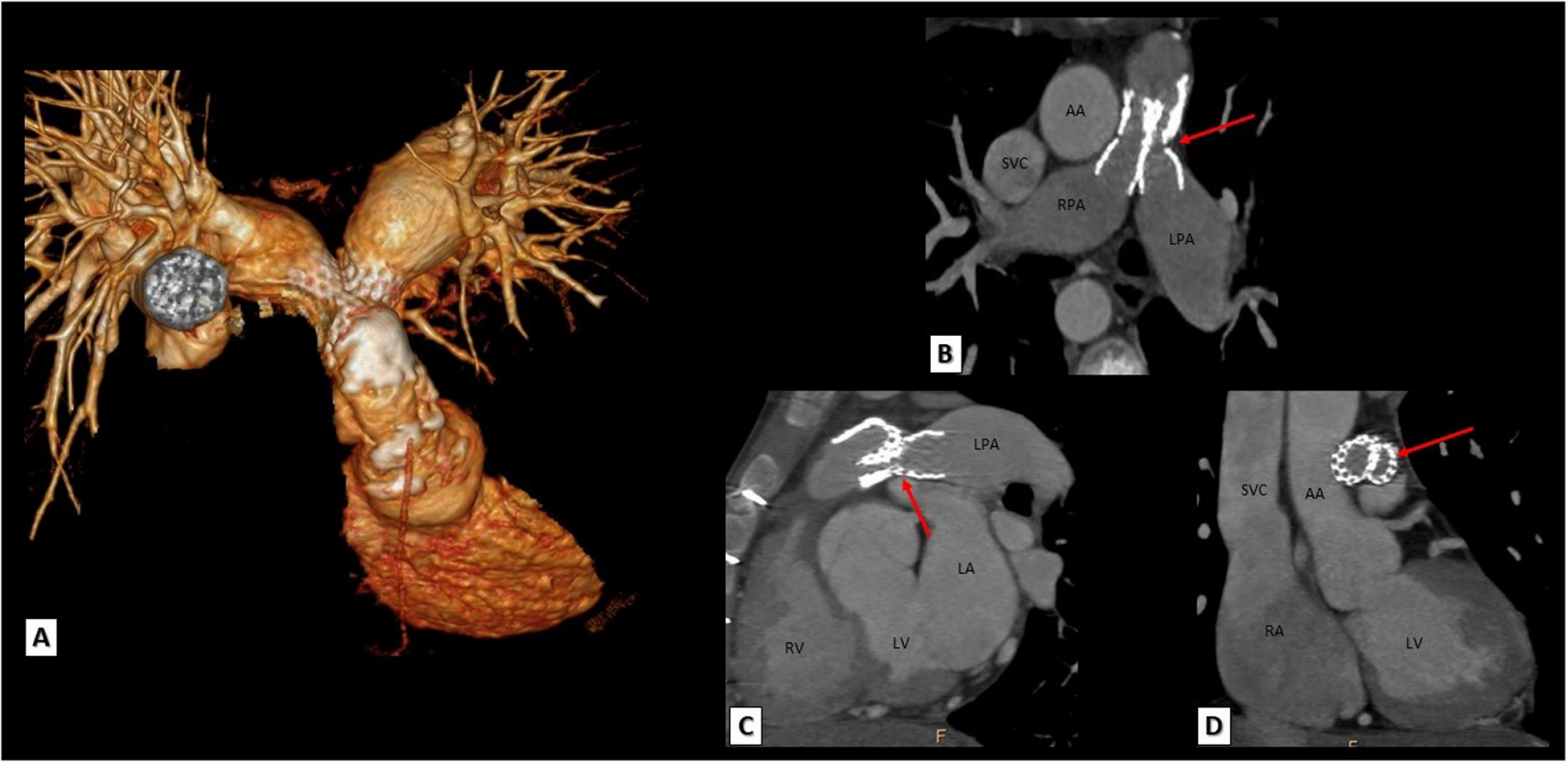
A TOF patient with both right and left pulmonary arteries stenting. **a** 3D VRT, **b** reformatted axial, **c** reconstructed sagittal, and **d** reconstructed coronal images showing LPA stent fracture with significant narrowing of LPA ostium (red arrows). AA, ascending aorta; RPA, right pulmonary artery; LPA, left pulmonary artery; SVC, superior vena cava; LV, left ventricle; RV, right ventricle; LA, left atrium; RA, right atrium

Palliative systemic to pulmonary shunts are usually performed in patients with small or hypoplastic pulmonary arteries in order to increase the pulmonary blood flow and thus promote the growth of pulmonary arteries. Evaluation of these shunts is an important indication of MDCT which can accurately evaluate shunt patency and any stenotic or occluded segments (Figs. 9, 10, and 11). As an alternative to surgical palliation, some selected patients with favorable anatomy may undergo percutaneous stenting of RVOT, PDA, or branch pulmonary arteries. MDCT is essential for evaluation of endovascular stents and can reliably evaluate the patency and position of these stents (Fig. 12).

### MDCT in surgically repaired TOF patients

#### Pulmonary regurgitation (PR) and RV dilatation

PR is one of the most commonly encountered complications following TOF repair. It is typically well tolerated in the short term due to RV hypertrophy which adapts the volume overload caused by PR. However, long-standing chronic PR can result in RV dilatation and failure, increasing tricuspid valve regurgitation, impaired exercise performance, supraventricular or ventricular arrhythmias, and sudden death [31]. MDCT is excellent in depicting the morphology of the RVOT and cardiac abnormalities related to PR in addition to accurate measurements of enlarged RV volumes which serve as one of the major criteria for PV replacement.

#### RVOT aneurysm

**Fig. 13:**
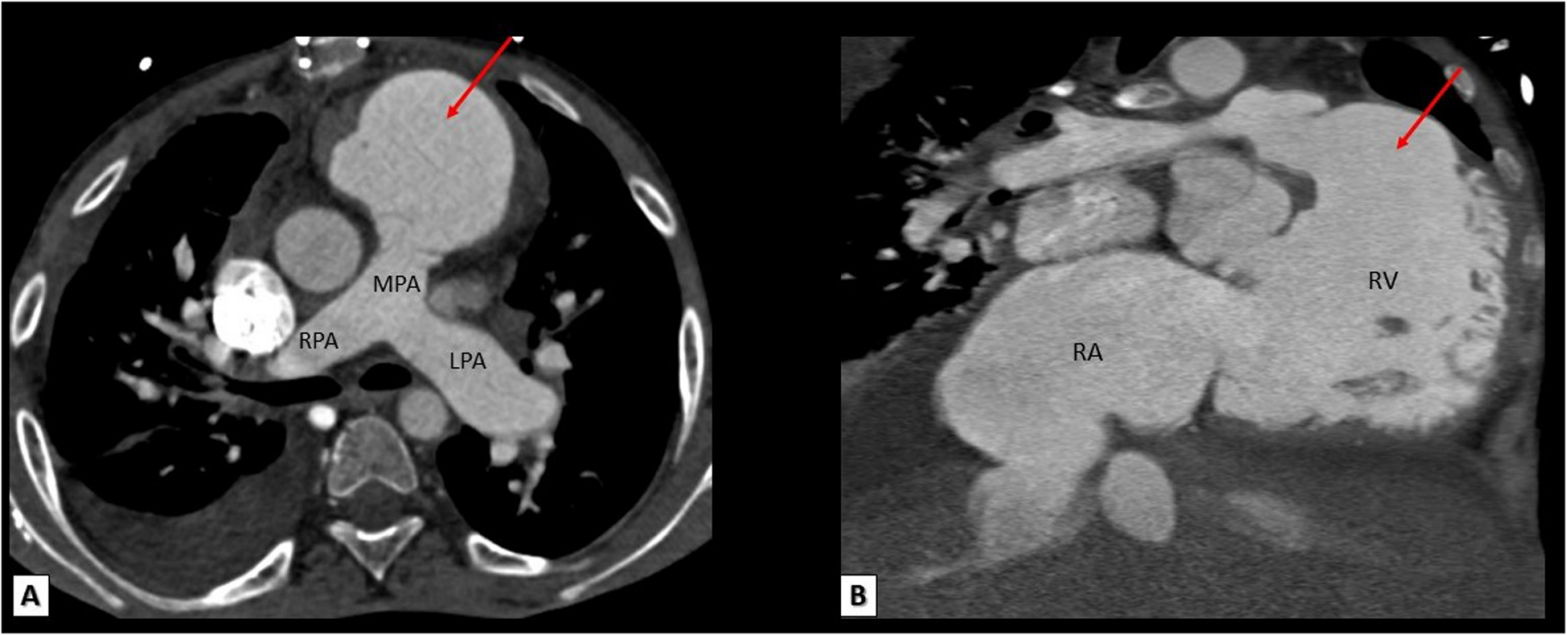
A surgically repaired TOF patient. **a** Reformatted axial and **b** reformatted coronal MDCT images showing a large RVOT aneurysm (red arrows). MPA, main pulmonary artery; RPA, right pulmonary artery; LPA, left pulmonary artery; RV, right ventricle; RA, right atrium

RVOT aneurysm is defined as outward movement of part of the ventricular wall or its repaired outflow tract during systole which is reported to be an independent predictor of RV dilatation and systolic dysfunction in repaired TOF patients. It also provides a suitable substrate for ventricular arrhythmias. MDCT images clearly delineate RVOT aneurysms and any associated dilatation of main pulmonary artery and its central branches (Fig. 13).

#### Conduit calcification and stenosis:

**Fig. 14:**
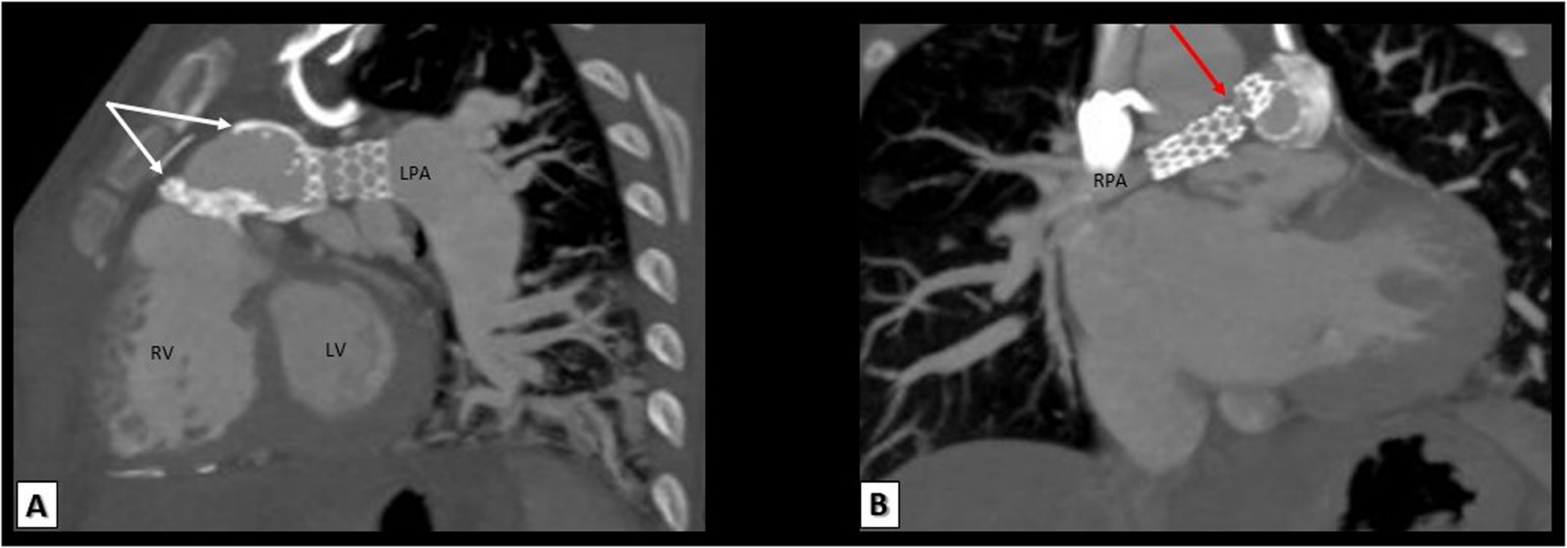
Images of a surgically repaired TOF patient with RV-PA conduit and bilateral branch pulmonary artery stenting. **a** Reconstructed sagittal and **b** reconstructed coronal MDCT images showing marked calcifications of the RV-PA conduit. Note the fractured RPA stent (red arrow). RPA, right pulmonary artery; LPA, left pulmonary artery; LV, left ventricle; RV, right ventricle

Different types of conduits are used in the surgical repair of TOF. The immediate postoperative results are excellent but with time, progressive conduit obstruction occurs due to patient-prosthesis mismatch, distal anastomotic stenosis, conduit kinking, thrombosis, and calcifications. MDCT can accurately assess the exact mechanism of conduit obstruction, as well as assessment of stenosis level, degree, and extension (Fig. 14).

## Conclusion

Because of its wide availability and high spatial and temporal resolution, MDCT is being used with increasing frequency to evaluate patients with TOF. It can reliably and accurately assess complex anatomy and associated anomalies in unrepaired TOF patients and guide the surgical approach and type of surgery needed. In addition, MDCT has given us the opportunity to fully understand and assess the late surgical sequalae, complications, and residual lesions. Therefore, it is now essential that corrected TOF patients should have regular follow-up to assess residual lesions and the development of these complications to manage them correctly.

## Data Availability

The datasets used during the current study are available from the corresponding author on reasonable request.

## Abbreviation

3D: Three-dimensional
4D: Four dimensional
ASD: Atrial septal defect
AVSD: Atrio-ventricular septal defect
CHD: Congenital heart disease
CMR: Cardiac magnetic resonance
DORV: Double orifice right ventricle
LAD: Left anterior descending
LM: Left main
LV: Left ventricle
MAPCA: Major aorto-pulmonary collateral artery
MBT: Modified Blalock-Taussig
MDCT: Multi-detector computed tomography
PDA: Patent ductus arteriosus
PFO: Patent foramen ovale
PG: Prostaglandin
PR: Pulmonary regurgitation
PV: Pulmonary valve
RCA: Right coronary artery
RV: Right ventricle
RVH: Right ventricular hypertrophy
RVOT: Right ventricle outflow tract
RVOTO: Right ventricle outflow tract obstruction
TOF: Tetralogy of Fallot
VSD: Ventricular septal defect
